# Acoustic emission of kidney stones: a medical adaptation of statistical breakdown mechanisms

**DOI:** 10.1007/s00240-024-01531-0

**Published:** 2024-02-20

**Authors:** Jack T. Eckstein, Oliver J. Wiseman, Michael A. Carpenter, Ekhard K. H. Salje

**Affiliations:** 1 Department of Earth Sciences, University of Cambridge, Downing St., Cambridge, Cambridgeshire CB2 3EQ UK; 2 Department of Urology, Cambridge University Hospitals NHS Foundation Trust, Hill’s Rd., Cambridge, Cambridgeshire CB2 0QQ UK

**Keywords:** Acoustic emission, Crackling noise, Urolithiasis, Fragmentation, Lithotripsy

## Abstract

Kidney stones have a prevalence rate of > 10% in some countries. There has been a significant increase in surgery to treat kidney stones over the last 10 years, and it is crucial that such techniques are as effective as possible, while limiting complications. A selection of kidney stones with different chemical and structural properties were subjected to compression. Under compression, they emit acoustic signals called crackling noise. The variability of the crackling noise was surprisingly great comparing weddellite, cystine and uric acid stones. Two types of signals were found in all stones. At high energies of the emitted sound waves, we found avalanche behaviour, while all stones also showed signals of local, uncorrelated collapse. These two types of events are called ‘wild’ for avalanches and ‘mild’ for uncorrelated events. The key observation is that the crossover from mild to wild collapse events differs greatly between different stones. Weddellite showed brittle collapse, extremely low crossover energies (< 5 aJ) and wild avalanches over 6 orders of magnitude. In cystine and uric acid stones, the collapse was more complicated with a dominance of local “mild” breakings, although they all contained some stress-induced collective avalanches. Cystine stones had high crossover energies, typically $\sim$ 750 aJ, and a narrow window over which they showed wild avalanches. Uric acid stones gave moderate values of crossover energies, $\sim$ 200 aJ, and wild avalanche behaviour for $\sim$ 3 orders of magnitude. Further research extended to all stone types, and measurement of stone responses to different lithotripsy strategies, will assist in optimisation of settings of the laser and other lithotripsy devices to insight fragmentation by targeting the ‘wild’ avalanche regime.

## Introduction

The prevalence and incidence of urolithiasis has increased dramatically over the last decade. This is a result of several factors, including increasing rates of obesity, global warming, and dietary changes [1, 2]. After several consecutive years of increase, patient episodes relating to upper urinary tract stones appear to be plateauing slowly [3]. This is despite the rise in metabolic syndrome, which increases with obesity and age [4].

Pathways for the management of urolithiasis have been laid down in guidelines issued by the European Association of Urology [5]. The recommended treatments are dependent on several factors, primarily size and location, with the three main interventions being extracorporeal shock wave lithotripsy (ESWL), retrograde intrarenal surgery (RIRS), and percutaneous nephrolithotomy (PCNL) [5, 6]. The parameters used during ESWL can be altered, according to changes seen in the stone and patient discomfort if the procedure is not undertaken with anaesthesia. The lithotripsy device used most commonly for RIRS is a laser, and two different lasers are currently in generalised use for this purpose: the holmium-YAG laser and the thulium fibre laser (TFL) [7, 8]. The energy, frequency, pulse width and modulation settings of these lasers can be changed according to desired effect and response of the stone. In PCNL, while laser energy can also be used, it is more common for ultrasound or ballistic energy probes to be used, sometimes alone and sometimes in combination.

Kidney stones are composed of a variety of different constituents [9–12] with the biochemical composition of the stone determining how they respond to different laser or lithotripter settings [7, 13]. Optimising the settings of devices which are used in the surgical treatment of stones may help to limit the operating time, as well as the energy needed to break a stone. These may lead to a reduction in the risk of complications for patients [14]. Using laser settings with excessive power may lead to excess heat generation in the kidney [15] with potential deleterious effects, such as has been reported in the initial experience with the TFL [16, 17]. An increase in operating time, apart from being associated with an increased cost to healthcare systems, is also associated with an increase in complications, such as sepsis [18].

Understanding the optimum operating conditions that lead to the most efficient stone fragmentation depending on stone type, whether for a laser, ultrasound, lithoclast or dual-energy device, would have significant benefits. At present, the chosen settings are often arrived at by trial and error, with starting settings altered according to the response of the stone, and the desired effect. In the case of laser use, this would be either dusting or fragmentation of the stone, or a combination of the two.

In a quite different field, there have been significant advances in understanding the physics of mechanical failure of porous media through analysis by acoustic emission (AE) spectroscopy of crackling sounds that are emitted during deformation under an external load. It has been found that crack propagation in materials as diverse as sandstone and sugar cubes occurs as a series of jerks with statistical patterns of amplitudes, energies and durations which are analogous to those that apply also, for example, in snow avalanches, metallic alloys, ice, crumpling of paper and the pathways of lightning flashes during storms [19–24]. A phenomenological treatment of overall mechanical deformation in terms of avalanches of acoustic emission signals yields values for fit parameters, such as power-law exponents and crossover energies between different power-law regimes, which provide quantitative measures of the failure behaviour of individual materials. The formal background to this approach has been set out in Salje et al. [19] and its applicability to the mechanical properties of kidney stones has been demonstrated in a preliminary study reported by Eckstein et al. [20].

The purpose of the present study was to use AE spectroscopy to investigate the variability of structural properties of the three principle types of kidney stones, namely weddellite, cystine and uric acid. Experimental evidence is constructed from the cracking of seven stones from each group under uniaxial stress. It is intended that the results should provide new and quantitative information relating to the mechanical stability of kidney stones that will help to inform medical professionals when choosing settings for their chosen lithotripsy device, and to aid the development of kidney stone removal technologies.

## Materials and methods

21 kidney stones fragments (7 fragments for each of the 3 stone types) removed, and categorised at the time of PCNL (Addenbrookes Hospital, Hills Rd, Cambridge CB2 0QQ), after ethical committee approval from the Cambridge human biology research ethics committee, were selected to represent three principal types. Samples A1–A7 were weddellite types IIa, IIb and IIc, B1–B7 were classified as cystine stones type Vb and C1–C7 were uric acid type IIIc according to the Daudon classification system [25]. Pictures of each sample are provided in Table 3 in the appendix.

**Fig. 1:**
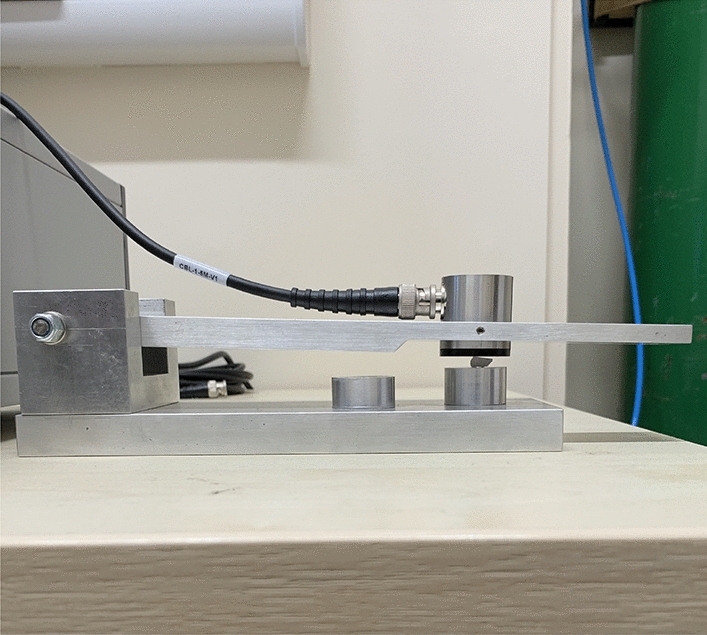
Experimental arrangement showing the press with the acoustic emission detector fastened on the top plate, in direct contact with the sample surface

Individual stones were loaded into a mechanical press where they were compressed with a piezoelectric transducer (Vallen-Systeme GmbH) in direct contact with the sample surface as shown in Fig. 1.

Before the start of each experiment, a noise measurement was performed for 10 min to determine the correct threshold for each sample. This eliminated the effect of environmental noise in the laboratory. The emitted acoustic emissions were transferred to the AMSY-6 measurement system (Vallen-Systeme GmbH). The crackling noise signals were then measured in terms of their energy in attojoule (aJ, 1 atto Joule is $10^{-18}$ Joule, the typical resolution of this technique is near 0.1 aJ) or their amplitude (ca. 8 $\upmu$V). The number of signals observed during compression was normalised so that they represented the probability distribution (number of signals with a given energy or amplitude/ total number of signals). This function is denoted as P(E) for the energy distribution and P(A) for the amplitude distribution. These results were plotted on a double logarithmic scale [26]. Following the naming convention set out by Refs. [22, 27–31], we refer to “wild” and “mild” behaviour where “wild” refers to deformation which is accommodated through synchronised, self-organised events forming avalanches, and “mild” refers to un-correlated events that have log-normal distributions.

Compressing the sample generated ‘crackling noise’ resulting from nucleating and propagating cracks which emitted acoustic signals. These acoustic signals were analysed and related to the properties of the kidney stones. The A-series (weddellite stones) are composed of calcium oxalate dihydrate and is among the most common kidney stone types. This mineral is also commonly found as authogenic crystals in sea floor mud, peat bearing sediments, and calcite bearing lucastrine sediments [32]. The B-series is the densest, and consists of cystine. At normal urinary pH cystine is insoluble in urine and cystine crystals aggregate in the bladder and kidney to form stones. The C-series (uric acid stones) are formed when uric acid precipitates out of solution resulting from low urine pH [33].

Samples A1–A7 were analysed in the frequency range of 25–850 kHz with a time resolution of 50 ns and a threshold of 23.6 dB. The next set, B1–B7 were measured in the frequency range of 100–1800 kHz with a time resolution of 25 ns and a threshold of 23.6 dB. Initially, this group had been measured with the same acquisition parameters as the weddellite series but preliminary experiments revealed that the avalanches in cystine stones occur over much shorter time scales and require a finer time resolution. Therefore, the time resolution was increased from 50 to 25 ns. Samples C1–C7 were analysed with the same acquisition parameters as the previous set.

## Results

**Fig. 2:**
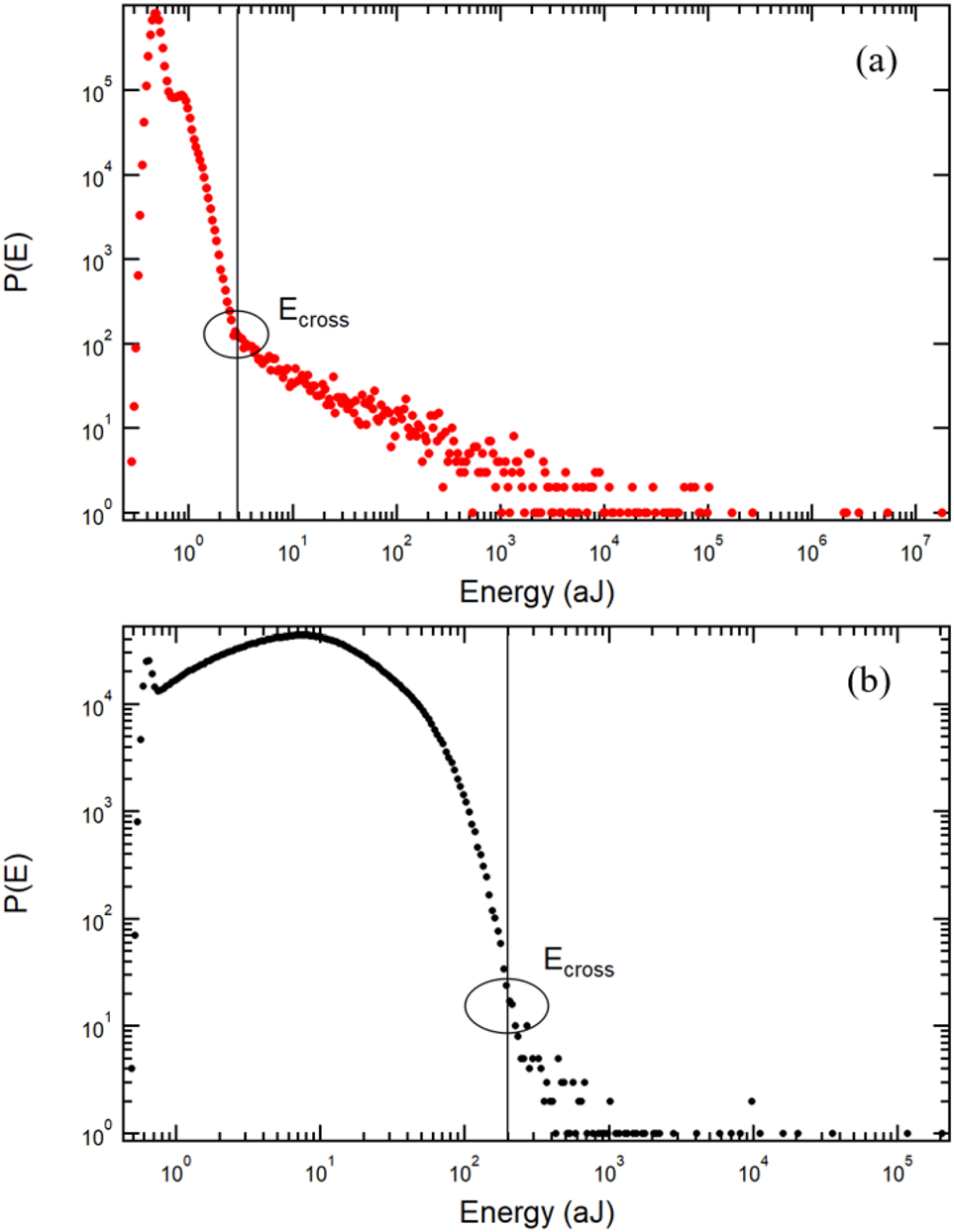
**a** Typical crackling noise distributions with power-law dependencies at high energies and a peak at low energies. The crossover energy is indicated by the vertical line. This was recorded from measurements on the weddellite stone (A-series). **b** Uric acid stone (C-series) shows mild signals below 210 aJ where the low-energy signals show a log-normal or Gamma function [26] while the high-energy signals follow a power law. Similar behaviour was found in the B (cystine) series but with extremely sparse signals in the power-law regime

The plots derived from our series of experiments are typically like the one shown in Fig. 2a. A very large peak occurs at low energies, indicating a large number of such events, and a much lower number of signals at higher energies. An important break of the functional distribution curves occurs in Fig. 2a at 3 aJ. At higher energies relating to ‘louder’ signals, we find a power-law dependence.

**Table 1:** Exponents for representative samples from the A (weddellite), B (cystine) and C (uric acid) series where $\epsilon$ is the energy exponent, $\tau$ is the amplitude exponent, $\alpha$ is the duration exponent, *X* is the amplitude—duration scaling exponent, $\chi$ is the energy—amplitude scaling exponent, and *D* is the value of duration

Parameter/sample	A2	B7	C2
$\epsilon$ (Energy)	1.55	1.3	1.5
$\tau$ (Amplitude)	2.1	1.6	2.0
$\alpha$ (Duration)	2.6	1.5 for $D\ge 10^4$ $\upmu$s	1.5 for $D\ge 10^4$ $\upmu$s
		2.5 for $D<10^4$ $\upmu$s	3.0 for $D<10^4$ $\upmu$s
*X* (Amplitude—duration)	1.3	1.5	1.5
	1.7		
$\chi$ (Energy—amplitude)	2	2	2

A mathematical description of the power-law exponents in the present table is given in Ref. [26]

Below the crossover at 3 aJ, the greater amount of signals peak near 0.6 aJ. The signals stem from local strain release under stress. Their two different fingerprints relate to random, independent events in the peak-regime at low energies and have correlated collapse by networks of cracks in the power-law regime [19, 34–37]. The boundary between the two regimes is referred to as the crossover point and separates the conditions where strain release occurs in a correlated manner (wild) and the low-energy regime where events are local and uncorrelated (mild) [38].

Figure 2b shows a similar curve of energy against probability for one of the C-series stones, composed of uric acid. It shows mild avalanche signals below 210 aJ where low-energy signals conform to a log-normal or Gamma function, while the high-energy signals show a power law. Similar behaviour was found in the B-series (cystine) but with extremely sparse signals in the power-law regime. The exponents can be extracted from the power-law regime and are shown in Table 1 for three representative stones (samples A2, B7, and C2) from each of the three stone classes examined in this study. Values for the crossover point ($E_\textrm{cross}$) from mild (uncorrelated), to wild (avalanche-like) for the all stones are given in Table 2.

**Table 2:** Avalanche exponent $\epsilon$ and crossover points between the mild and wild events for different stone fragments from the A-series (weddellite), B-series (cystine) and C-series (uric acid)

Sample/parameter	$\epsilon$	$E_\textrm{cross}$
A1	1.6 for $E<10^5$ aJ	3 aJ
	1.5 $E>10^5$ aJ	
A2	1.55 for $E<10^5$ aJ	3 aJ
	1.4 $E>10^5$ aJ	
A3	1.7 aJ	3.5 aJ
A4	1.57 for $E<10^5$ aJ	3 aJ
	1.40 $E>10^5$ aJ	
A5	1.55 for $E<10^4$ aJ	2.8 aJ
	1.35 $E>10^5$ aJ	
A6	1.53	2.5 aJ
A7	1.5 for $E<10^5$ aJ	2.5 aJ
	1.2 $E>10^5$ aJ	
B5	1.2	750 aJ
B7	1.3	700 aJ
C1	1.4	200 aJ
C2	1.5	250 aJ
C3	1.6 for $E<10^5$ aJ	200 aJ
	1.4 $E>10^5$ aJ	
C4	1.9 for $E<600$ aJ	200 aJ
	1.6 $E>600$ aJ	
C5	1.6	280 aJ
C6	1.6	200 aJ
C7	1.9 for $E<10^3$ aJ	210 aJ
	1.5 $E>10^3$ aJ	

Results from sample C4 have been discussed in detail in Ref. [20] and are reproduced in the present study with permission

### Energy and amplitude scaling

For the sake of simplicity, data sets for samples A2, B7, and C2 are displayed in Figs. 3, 4, 5, 6, 7 and 8 as being representative of each of the kidney stone types. Figures 9, 10, 11, 12, 13, 14, 15, 16, 17, 18, 19, 20, 21 and 22 in the appendix include additional energy probability distribution functions (PDFs) and maximum likelihood (ML) analysis which show the results for all samples summarised in Table 2. Crackling noise spectra, i.e. the experimental jerk spectra, for samples A2, B7 and C2 are shown in Fig. 3.

**Fig. 3:**
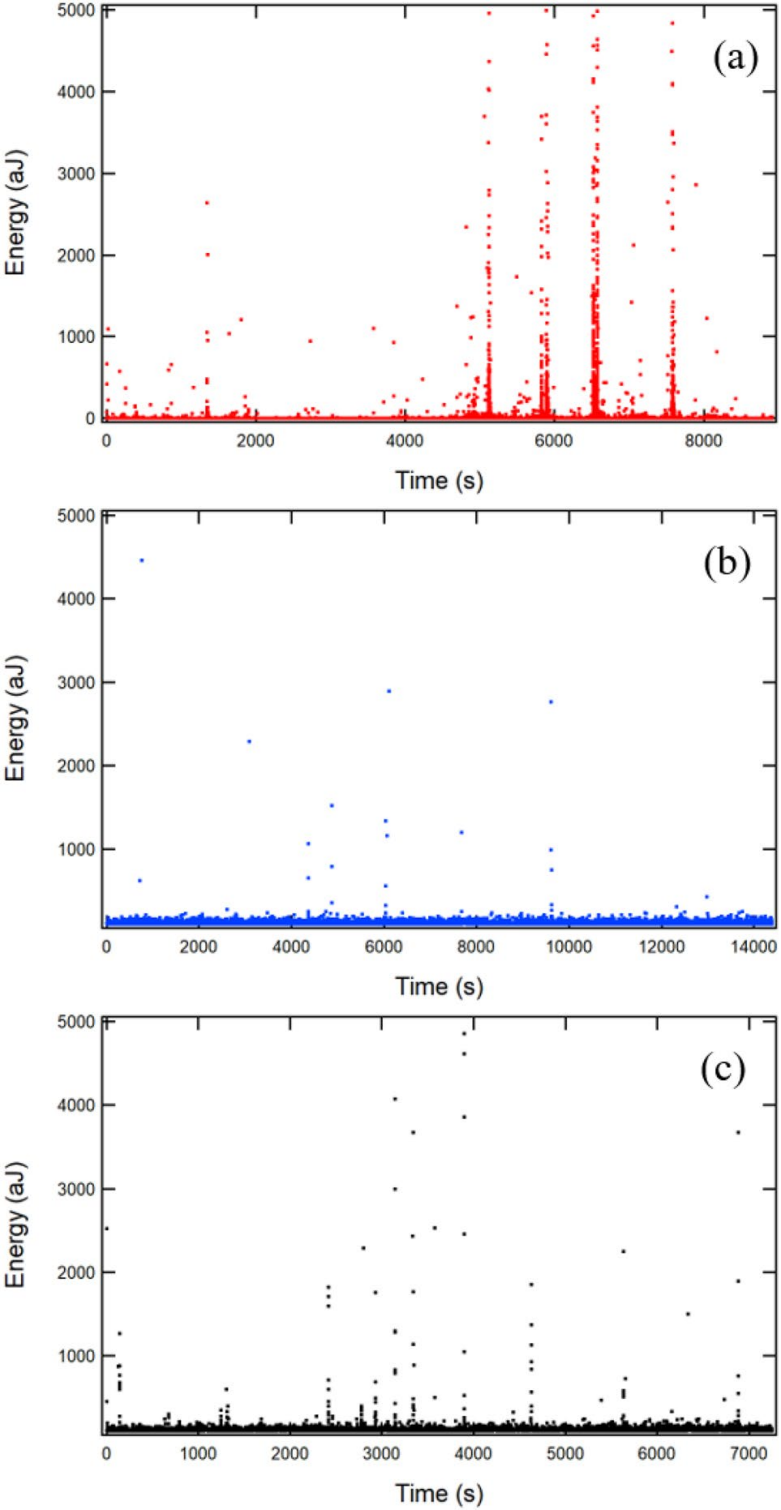
Energy jerk spectra for samples A2, weddellite (**a**), B7, cystine (**b**), and C2, uric acid (**c**) showing energy on the *y*-axis (aJ) vs time (s) on the *x*-axis

The jerk distribution is unusual for typical crack propagation in minerals where the density of jerks per time interval is fairly constant. The ‘standard’ behaviour is classified as ‘stationary’ because the number of events per time interval is roughly constant throughout the experiment [39, 40]. In Fig. 3 the behaviour of our samples is shown to be non-stationary with some bursts separated by a multitude of very weak signals and quiet periods. This sets the scene for the discussion of avalanches: the big peaks are the wild avalanches (highly non-stationary) and the weak, stationary background signals indicate mild events. Furthermore, Fig. 3 shows that bursts of the kind shown in Fig. 3a are slightly more numerous with shorter intervals in between (i.e. slightly more stationary) than in Fig. 3b and Fig. 3c. The bursts in Fig. 3b are extremely sparse and non-stationary, while the low-energy signals are abundant and stationary. We find an extreme predominance of mild events in this case.

**Fig. 4:**
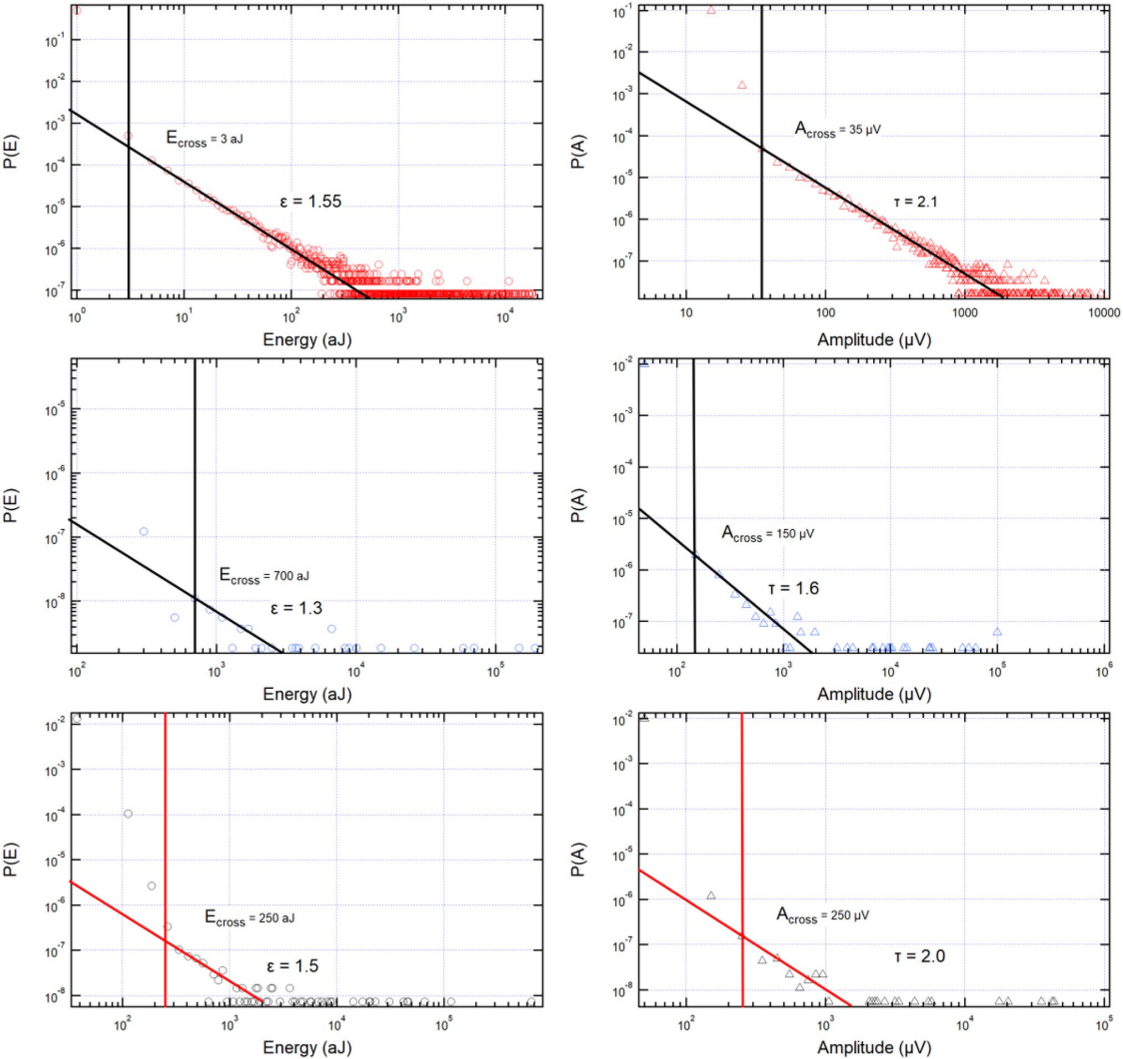
Energy and amplitude probability distribution functions highlighting the power-law regime, i.e. for $E \ge E_\textrm{cross}$ and $A \ge A_\textrm{cross}$ for the representative stones from the A-series weddellite stones (**a**, **b**), B-series cystine stones (**c**, **d**), and C-series uric acid stones (**e**, **f**). The energy and amplitude crossovers are 3 aJ and 35 $\upmu$V for sample A2, 700 aJ and 150 $\mu$V for B7, and 250 aJ and 250 $\mu$V for C2. The energy exponents for samples A2, B7 and C2 are $\epsilon = 1.55$. $\epsilon = 1.3$, and $\epsilon = 1.5$, respectively. The amplitude exponents are $\tau = 2.1$, $\tau = 1.6$, and $\tau = 2.0$, respectively

The jerk spectra show typical characteristics which are quantified in Fig. 4. The wild, power-law distributed region is marked by large jerks and are dominant in the A-series, (weddellite). The power-law regime is much shorter in the B (cystine)- and C (uric acid)-series where the mild events dominate. The crossover points between mild and wild events are indicated by vertical black lines in Fig. 4. A low crossover energy means that the collapse occurs predominantly by collective avalanches, which are typical for hard brittle materials, while a high crossover energy means that the dominant collapse mechanism is by mild events, similar to the behaviour of soft, non-brittle materials.

**Fig. 5:**
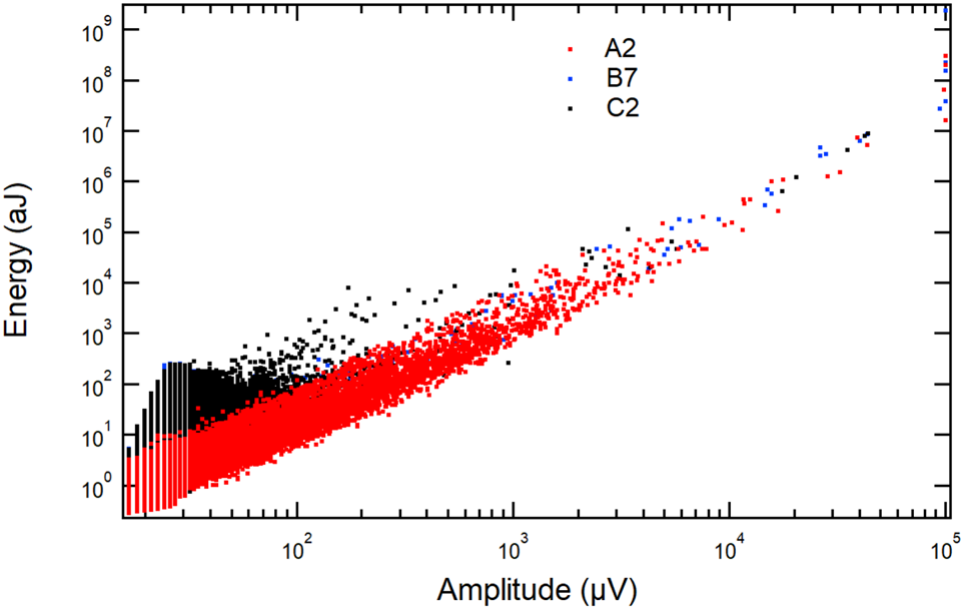
Energy and amplitude scaling for each of the representative kidney stones showing the approximate scaling $E\propto A^2$. Sample A2 is shown in red, B7 in blue and C2 in black

The probability distributions in Fig. 4a (A-series) range from 1 to 10,000 aJ. The B- and C-series have shorter power-law regimes and the determination of the exponents is difficult, comparatively. The amplitudes and the energies of wild avalanches are highly correlated. Depending on the waveform of the emitted signal, the most common correlation is $E \propto SA^x$, where the exponent *x* takes values around 2. The correlation between energies and amplitudes of all series follows the predicted scaling $E \propto A^2$ rather well (Fig. 5) [41] with ca. 6 orders of magnitude of the power laws in the A-series.

The scaling prefactor *S* varies between the data sets when very different mechanisms are superimposed. This effect is called multi-branching and has been commonly observed, for example, in Refs. [21, 42, 43].

**Fig. 6:**
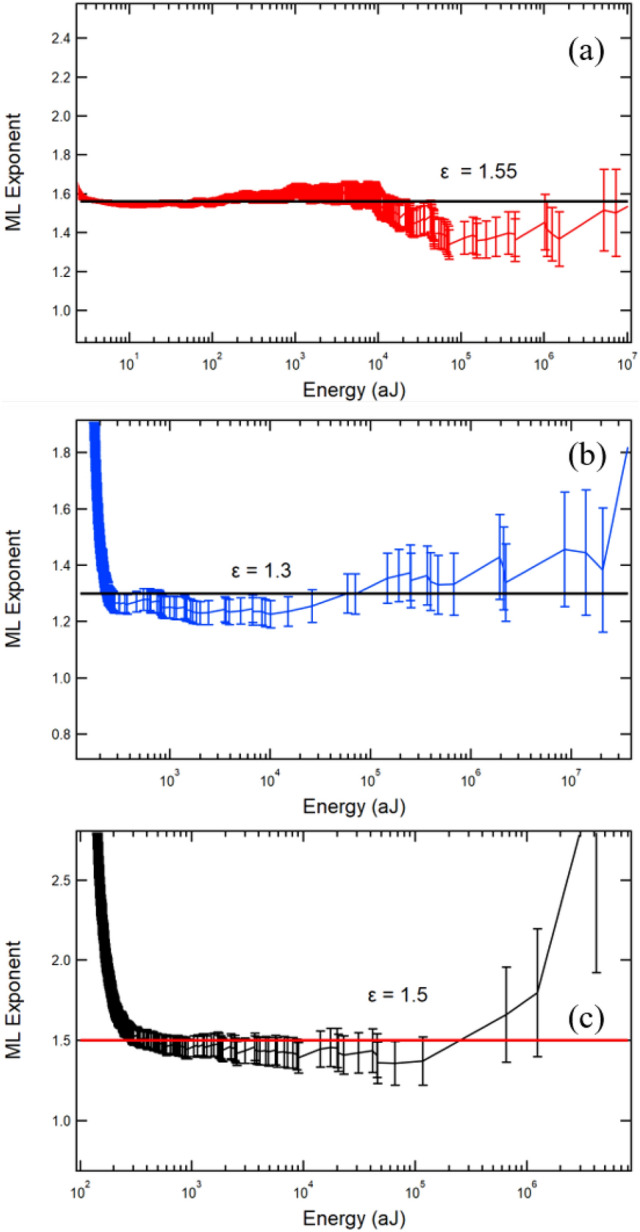
Maximum likelihood analysis of samples A2 (**a**), B7 (**b**), and C2 (**c**) showing energy exponents of $\epsilon =1.55$, $\epsilon =1.3$, and $\epsilon =1.5$, respectively

In Fig. 6 we show the correlation curve which indicates that the scaling is approximately uniform and, therefore, multiple collapse mechanisms do not contribute significantly to the overall signal. The variation which is observed is the same for collapse mechanisms under compression in vastly different materials. An advanced method to display the power-law behaviour is the maximum likelihood (ML) estimate [44, 45]. If the AE parameter is power-law distributed, the ML curve shows a plateau, the value of which is equal to the power-law exponent [46].

Figure 6a shows the largest plateau over seven decades, observed in weddellite stones. The exponent is $\epsilon = 1.55$. The B-series has a slightly lower exponent of $\epsilon = 1.3$, which is remarkably close to the ‘critical mean field’ value of 1.33 [26]. Despite the sparse statistics, the B-series samples B7 and B5 provide sufficiently good data to perform statistical analysis. The length of the plateau is surprisingly large so that the energy exponents are fully confirmed. The results for the wild avalanches are summarised in Table 1. The estimated errors are $\pm {0.1}$. The self-consistency of the exponents is confirmed by the scaling relationship [26] $2(\epsilon -1) = (\tau -1)$ in mean field theory. Our experimental results fulfil this relationship reasonably well.

### Duration and cross-scaling

Wild avalanches take some time to develop. The duration of an avalanche is defined as the total time during which an avalanche persists. It is measured in an AE experiment between the first threshold crossing of an acoustic signal, and the last threshold crossing of the emitted acoustic wave. The points shown in Fig. 3 are examples of classic singular acoustic emissions. The distribution of avalanche durations is also a power law with an exponent $\alpha$. However, while the duration statistics are commonly well defined, kidney stones of the B- and C-series prove problematic. The origin of this difficulty is explained in the present section.

**Fig. 7:**
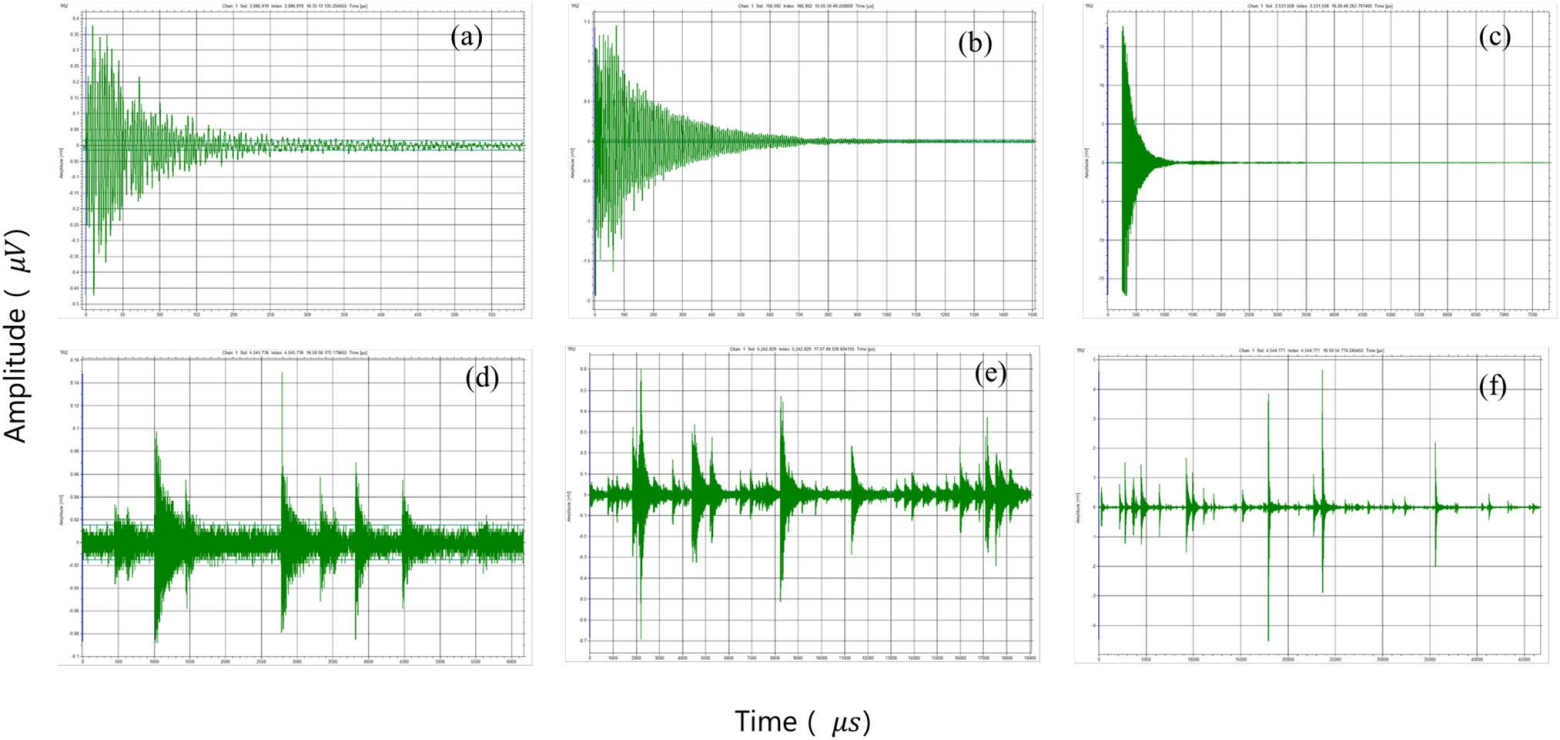
Individual waveforms from sample A2 along the line $X = 1.3$ (**a**–**c**), and $X = 1.7$ (**d**, **e**). The peak amplitude and durations are: **a** $A = 474.9$ $\upmu$V, $D = 588.0$ $\upmu$s; **b** $A = 1942.4$ $\upmu$V, $D = 1502.5$ $\upmu$s; **c** $A = 17,734.0$ $\upmu$V, $D = 7786.7$ $\upmu$s; **d** $A = 150.7$ $\upmu$V, $D = 6162.1$ $\upmu$s; **e** $A = 692.6$ $\upmu$V, $D = 19,056.9$ $\mu$s; and **f** $A = 4644.4$ $\upmu$V, $D = 46,684.1$ $\upmu$s

Figure 7a–c shows classical acoustic emissions. However, avalanches can generate aftershocks, which means multiple acoustic waves will not cross the minimum threshold resulting in an inflated duration as shown in Fig. 7d, e. Furthermore, avalanches overlap and individual avalanches are hard to identify. Figure 7d–f, shows typical spectra with multiple avalanches, where individual points on the energy spectra should be treated as an event with a short duration. However, because there is no minimum threshold crossing, multiple avalanches are bundled together resulting in very high nominal values of their duration. Samples A2 and A3 have fewer bundled avalanches with an exponent of $\alpha \approx 2$. The B- and C-series have many avalanche bundles. Samples C3 and C4 with duration exponents of $\alpha =2.3$, and $\alpha =2.8$, respectively, are typical for wild avalanches. Lower values near 1.5 represent bundled avalanches of the type shown in Fig. 7d, e.

**Fig. 8:**
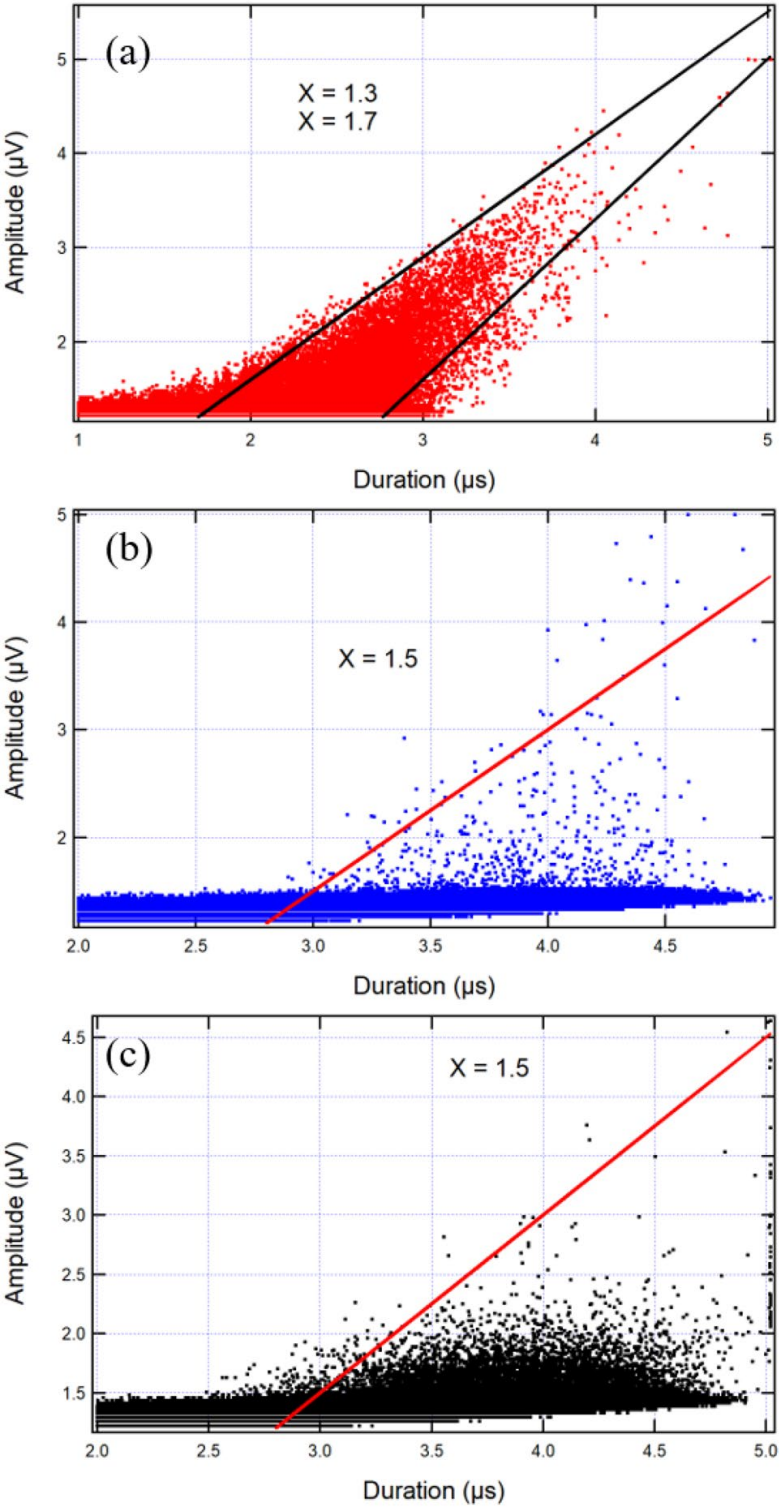
Amplitude–duration scaling revealing exponents $X=1.3$, and $X=1.7$ for A2 (**a**), $X = 1.5$ for B7 (**b**), and $X = 1.5$ for C2 (**c**)

A more obvious way to present this phenomenon is to plot the cross correlation between durations and amplitudes (or energies) of wild avalanches as shown in Fig. 8. The scaling relationship shows correlation exponents of amplitudes versus durations near 1.5, which is the theoretical value for wild avalanches [21]. However, the data are very noisy because the mild events strongly interfere with the jerk distributions. The distribution is somewhat constrained in Fig. 8a and almost random in Fig. 8b, c. On closer inspection, we find that all data with higher durations than those indicated by the lines are bundles and, hence, artificially long. Conversely, Fig. 7a–c shows single events following points along the line $X = 1.3$ in Fig. 8a.

**Fig. 9:**
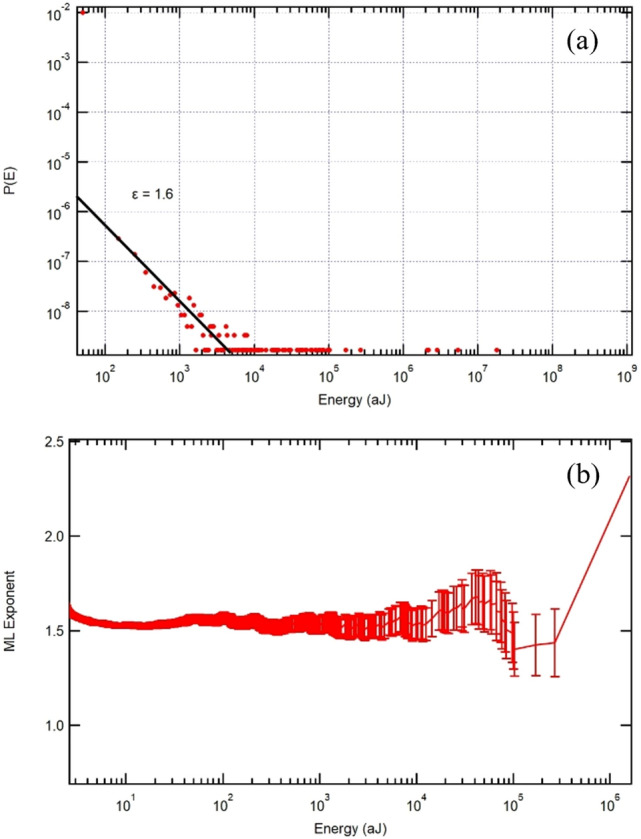
Energy PDF (**a**) and ML (**b**) for sample A1 revealing an energy exponent of $\epsilon =1.6$ over four decades

**Fig. 10:**
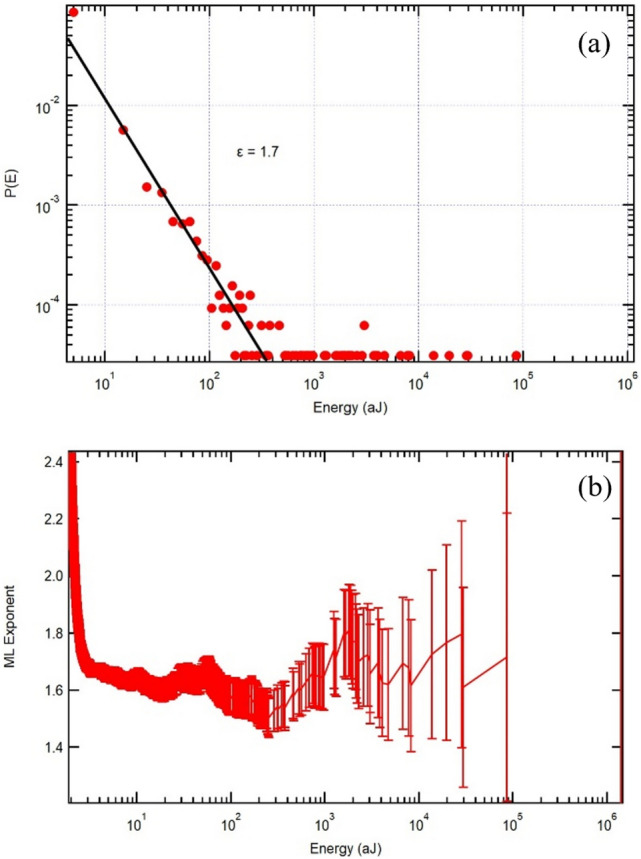
Energy PDF (**a**) and ML (**b**) for sample A3 revealing an energy exponent of $\epsilon =1.7$ between 500 and 50 aJ. The ML reveals an increase in $\epsilon$ to $\sim 1.8$

**Fig. 11:**
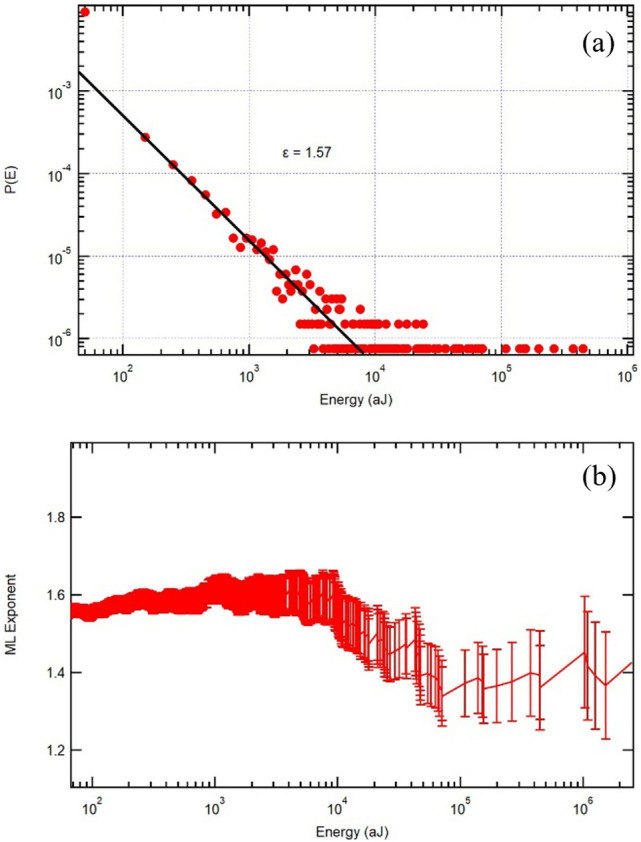
Energy PDF (**a**) and ML (**b**) for sample A4 revealing an energy exponent of $\epsilon =1.57$ between 100 and 100,000 aJ. Between $10^4$ and $10^5$ aJ there is a continuous decrease in $\epsilon$ from 1.6 to 1.4

**Fig. 12:**
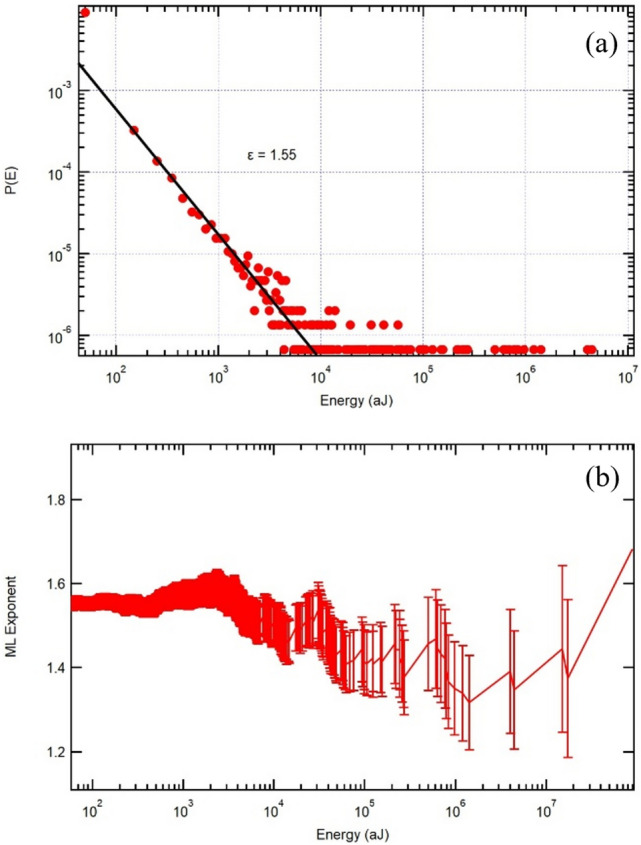
Energy PDF (**a**) and ML (**b**) for sample A5 revealing an energy exponent of $\epsilon =1.55$ between 100 and 100,000 aJ. Between $10^3$ and $10^7$ aJ there is a gradual decrease in $\epsilon$ from 1.55 to 1.35

**Fig. 13:**
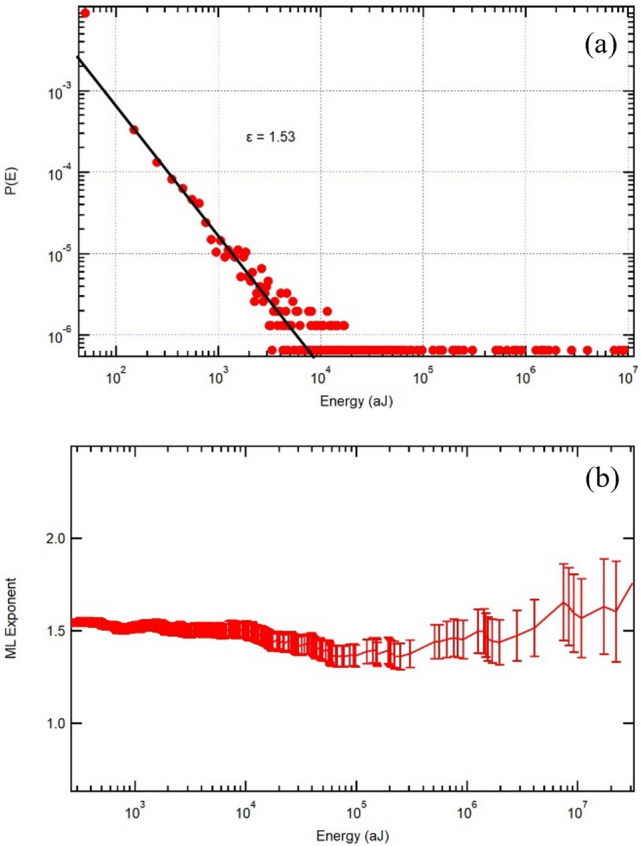
Energy PDF (**a**) and ML (**b**) for sample A6 revealing an energy exponent of $\epsilon =1.53$ stable over four decades. There is however, a subtle decrease in $\epsilon$ from 1.53 to 1.5 at $10^4$ aJ

**Fig. 14:**
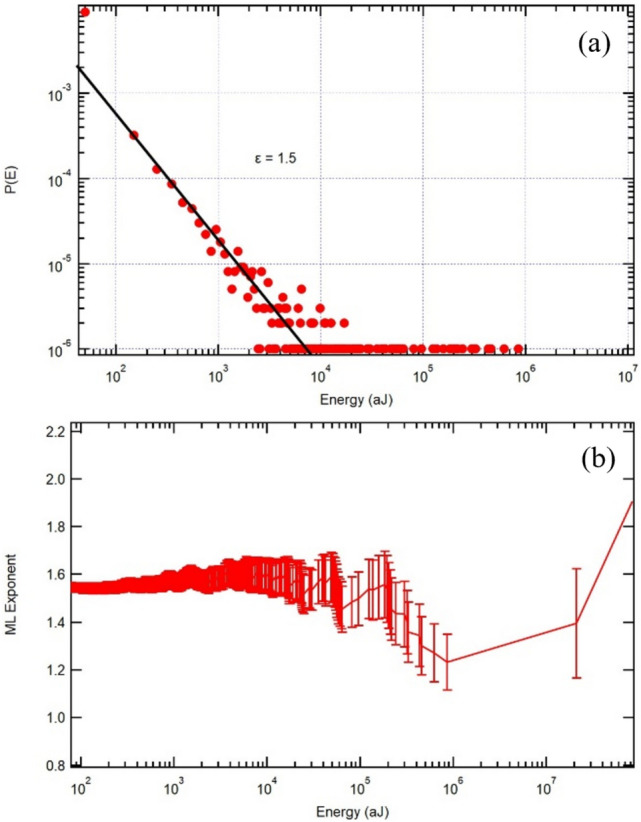
Energy PDF (**a**) and ML (**b**) for sample A7 revealing an energy exponent of $\epsilon =1.5$ stable over three decades. There is a sharp decrease in $\epsilon$ from 1.5 to 1.2 between $10^5$ and $10^6$ aJ

Data for sample A1 in Fig. 9 reveal an energy exponent of $\epsilon = 1.6$ over four decades with a small hump and slight reduction in $\epsilon$ to 1.5 at $10^5$ aJ. Sample A2 has an energy exponent of $\epsilon = 1.55$ over four decades and reduces to $\sim 1.4$ again at $\sim 10^5$ aJ (Fig. 5b). Sample A3 has some oscillations but in the low-energy regime is stable at $\epsilon =1.7$ between 500 and 50 aJ. Upon increasing energy, there is an increase of $\epsilon$ to 1.8; however, at energies greater than $10^4$ aJ the error increases to ± 0.25, as shown in Fig. 10, making it difficult to determine if the higher energy exponent is meaningful. Sample A4 has an energy exponent of $\epsilon =1.57$ between 100 and 100,000 aJ. Between $10^4$ and $10^5$ aJ, there is a continuous decrease in $\epsilon$ from 1.6 to 1.4 shown in Fig. 11. Sample A5 has an energy exponent of $\epsilon =1.55$ between 100 and 100,000 aJ (Fig. 12). Between $10^3$ and $10^7$ aJ, there is a gradual decrease in $\epsilon$ from 1.55 to 1.35. Figure 13 shows energy PDF and ML for sample A6 revealing an energy exponent of $\epsilon =1.53$ stable over four decades. There is, however, a subtle decrease in $\epsilon$ from 1.53 to 1.5 at $10^4$ aJ. Sample A7 has an energy exponent of $\epsilon =1.5$ stable over three decades. There is a sharp decrease in $\epsilon$ from 1.5 to 1.2 between $10^5$ and $10^6$ aJ (Fig. 14).

**Fig. 15:**
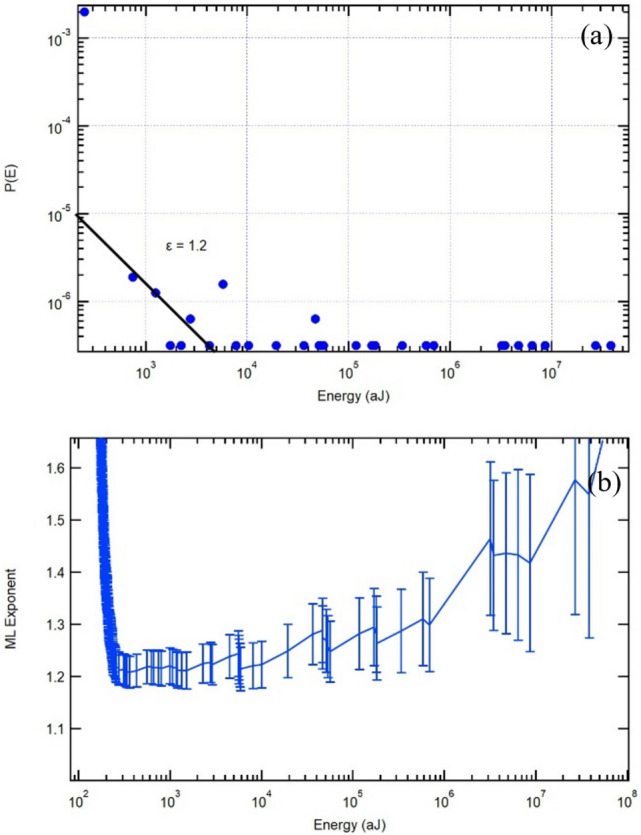
Energy PDF (**a**) and ML (**b**) for sample B5 revealing an energy exponent of $\epsilon =1.2$ stable over a narrow region at low energies. The ML mimics the behaviour seen in exponentially damped power-law systems and is marked by a gradual increase in $\epsilon$ as the energy increases

The only experiments in the B-series that generated enough data to perform meaningful statistics were from samples B5 and B7. This is a result of the much finer time scales over which the cystine stones generated AEs. They occurred at some duration less than the limit of detection, i.e. $< 25$ ns, owing to the sparse data sets in comparison to the weddellite, and uric acid stones. Sample B5 gave an exponent of $\epsilon =1.2$ (Fig. 15) and was stable over a narrow region at low energies. The ML mimics the behaviour seen in exponentially damped power-law systems [44, 47–49] and is marked by a gradual increase in $\epsilon$ as the energy increases. The same dynamics are seen in sample B7, except the exponent is 1.3 and it is stable over a slightly larger energy range between $10^3$–$10^4$ aJ before continually increasing. This indicates that there was only one mechanism, and it occurred over short time scales ($D < 50$ ns; most likely crack propagation).

**Fig. 16:**
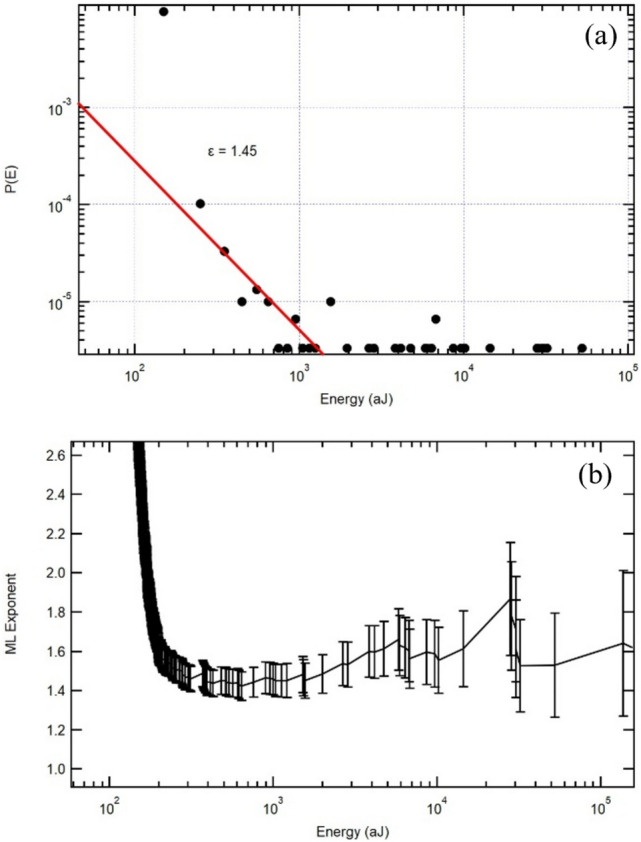
Energy PDF (**a**) and ML (**b**) for sample C1 revealing an energy exponent of $\epsilon =1.45$ stable between 250 and 1000 aJ. For energy values greater than 1000 aJ the energy exponent gradually increases to 1.6 at $E = 5500$ aJ but reduces again to 1.4 at  12,000 aJ

**Fig. 19:**
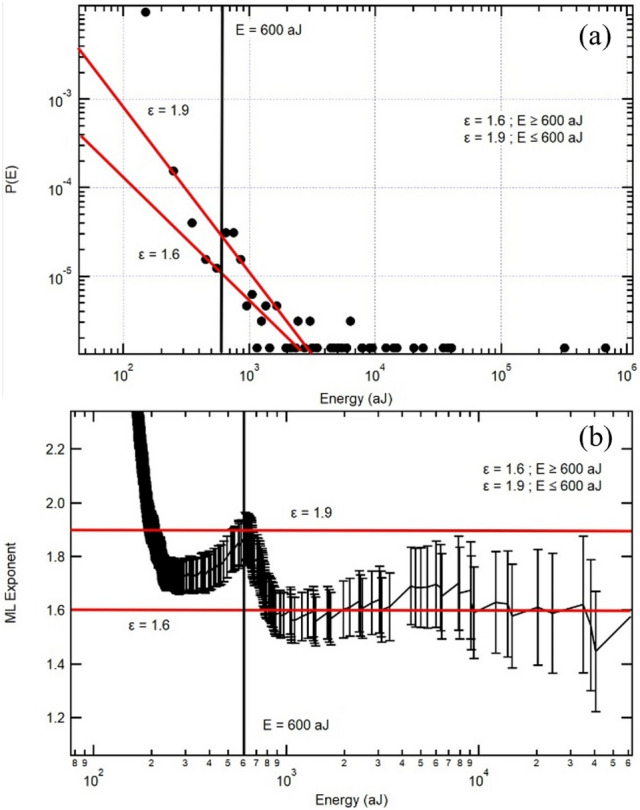
Energy PDF (**a**) and ML (**b**) for sample C4 revealing two energy exponents of $\epsilon =1.9$, and $\epsilon = 1.6$. For energies larger than 600 aJ $\epsilon =1.6$ and for energies less than 600 and greater than 30 aJ there is a sharp increase in energy exponent reaching a maximum of $\epsilon =1.9$. Reproduced from Ref. [20]

**Fig. 22:**
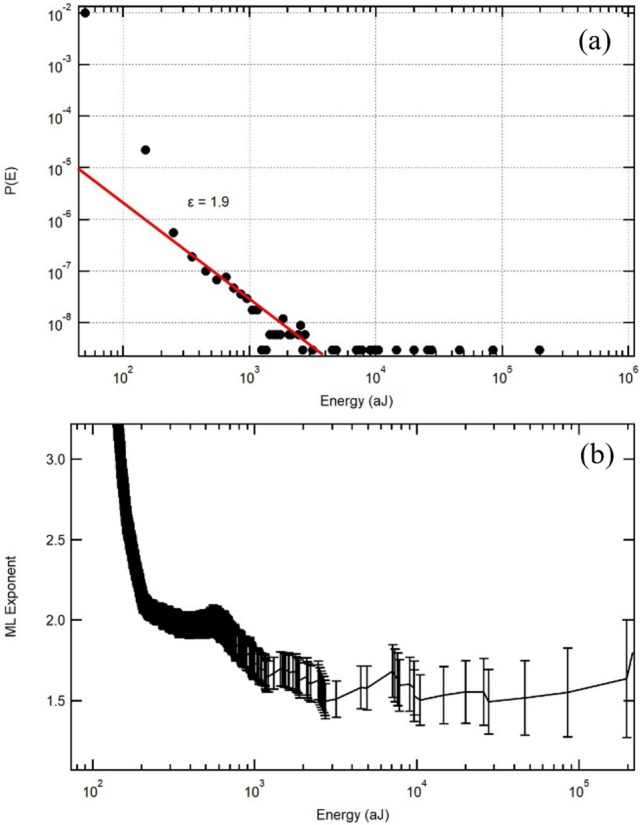
Energy PDF (**a**) and ML (**b**) for sample C7 revealing two energy exponents of $\epsilon =1.9$ for $E<10^3$, and $\epsilon =1.5$ for $E>10^3$

Figure 16 shows energy PDF and ML for sample C1 revealing an energy exponent of $\epsilon = 1.45$ stable between 250 and 1000 aJ. For energy values greater than 1000 aJ the energy exponent gradually increases to 1.6 at $E = 5500$ aJ but reduces again to 1.4 at 12,000 aJ. Sample C2 yielded an exponent of $\epsilon =1.5$ which is stable over the entire energy spectrum with no indication of multiple failure mechanisms at play. Sample C3 gave an energy exponent of $\epsilon =1.6$. There is a small hump in $\epsilon$ between 400 and 1,200 aJ and a decrease in $\epsilon$ from 1.6 to 1.4 at $10^5$ aJ. C4 gave perhaps the most obvious change in exponent, as shown in Fig. 19. $\epsilon =1.6$ for energies larger than 600 aJ, and there is a sharp increase in energy exponent reaching a maximum of $\epsilon =1.9$ for energies less than 600 and greater than 30 aJ. The results from kidney stone sample C4 were discussed in detail in Ref. [20]. C5 gave a relatively constant exponent of $\epsilon =1.6$ until $10^4$ aJ and there are no signs of multiple processes. Similarly, C6 gave only one exponent of $\epsilon =1.6$ and no AEs with $E>10^5$ aJ. This means that for this experiment, the low-energy failure mechanism was all that was needed to instigate failure. C7 gave two energy exponents of $\epsilon =1.9$ for $E<10^3$, and $\epsilon =1.5$ for $E>10^3$ (Fig. 22. A summary of these results is shown in Table 2.

The total number of uncorrelated events in our study was, in general, much greater than the correlated events where only the correlated events represent the breaking of the hard and brittle parts of the kidney stones. It is, therefore, important to distinguish between the mild events and wild avalanches very carefully. Note that this behaviour has been observed before in a few other materials [50] but it is certainly not typical for crack-induced collapse of materials. The jerk energies and amplitudes in the wild regime are power law distributed with energy and amplitude exponents $\epsilon$ and $\tau$: $P(E) \approx E^{-\epsilon }$ and $P(A) \approx A^{-\tau }$.

## Discussion

The key discovery of our study is that the mechanical collapse of kidney stones occurs with two distinct mechanisms, in conformance with the behaviour of many other materials subjected to compression. All the kidney stones showed avalanches and localised events which match with previous descriptions of wild avalanches and mild events [27, 31]. Wild avalanches show correlated cracking like teeth and most brittle materials [51–57]. Such ‘hard’ materials break by extended avalanches and complex crack patterns. Mild events are local and do not induce extended cracks in the same stones in response to wide ranging elastic forces [22, 30, 38, 58].

The proportion of wild and mild events is different for stones of differing mineral consistency, or type. However, it is worth noting that while stones are typically described as a single or mixed mineral type, in fact, they contain many organic substances, primarily proteins. The effect that these may have on collapse mechanisms, their variability between stones described as being of the same mineral component, and their spatial orientation leading to stone heterogeneity will all undoubtedly play a significant role in stone fragmentation [59–61]. Those of the A-series (weddellite) show a dominance of wild avalanches with similar characteristics to other brittle materials. The important parameter is the crossover point between wild and mild event, shown in Table 2.

The weddellite stones (A-series) showed a mixture of intergranular motion, porous collapse, and crack formation/propagation. The cystine stones (B-series) are the most dense and microscopy studies have revealed that the centre of these stones are homogeneous, and naturally form a hexagonal structure owing to their characteristic high density and mono-dominant failure mechanism [33]. The failure mechanism is a mixture of crack formation / propagation which happens on short time scales and emits high strain energies. In addition, they showed a large component of mild events with low emitted energies. The avalanches displayed no further energy exponent mixing lending support for there being only one dominant avalanche failure mechanism in addition to the mild events. They also gave the closest to the critical mean-field predicted value ($\epsilon = 1.33$) [26]. These observations agree with crack propagation observed in other materials [35, 62]. The C-series (uric acid stones) are more loosely packed and the main failure mechanism is intergranular failure. When considering the relative contributions of mild and wild “avalanche” collapse, there are orders of magnitude more mild events for all stone types, indicating that these events likely also play a major role in stone fragmentation.

The values of the crossover points in the weddellite stones are between 2.5 and 3 aJ. These are very low values which show that many events are wild, i.e. brittle behaviour dominates. When a crack is initiated, the crack propagates easily throughout the sample. The most striking observation was the wide energy range over which the weddellite stones showed power-law distributed events, and the extremely low energies for which the power law remains. This suggests that weddellite stones can fail at very low values of input energy if one can target the large range of energies over which the wild avalanches preside.

This result differs greatly from the results in the cystine stones. The crossover points were typically $\sim$ 750 aJ in this stone class. Very large values for the crossover point suggested very few correlated cracks occur. Instead, we observed that cracking mainly involved the removal of slightly attached small grains. This indicates that these grains were firmly attached and that large parts of the sample deformed without initiating correlated cracks throughout the sample. Very few avalanches occurred and the collapse was dominated by local breakings. Cystine stones showed high crossover points and a narrow avalanche regime (ca. 1 order of magnitude) which would make it difficult to target, and subsequently incite low-energy failure. However, as mentioned in the prior section, cystine stones emitted acoustic emissions on a much faster time scale compared to the weddellite and uric acid stones ( i.e. < 25 ns) which raises the suspicion that this class of stones may have a wider avalanche regime, but they occurred on time scales outside the limit of detection.

The uric acid stones behaved as an intermediate between the weddellite and cystine stones, with a crossover point of $\sim$ 250 aJ, i.e. much lower than the cystine stones, but higher than weddellite stones. When parts are broken, the sample is less strained and emits weaker signals. The small but well-developed avalanche regime (see Fig. 2b) shows well-defined exponents which are very close to the classic mean field behaviour. Therefore, it should be possible to target the range over which uric acid stones show power-law distributed events (ca. 3 orders of magnitude) to incite low-energy failure.

An understanding of the main ways in which different types of stones fragment should allow us to adapt the technologies we have to fragment stones, and to individualise the settings of these technologies for known stone types. The composition of stones may be known pre-operatively, from a previous stone analysis, for example, or from intra-operative identification of the stone by, for example, artificial intelligence and image processing techniques [63–72] or the application of an AFM probe at the start of surgery, can measure nanoscale crackling noise based on AFM nanoindentation, a technique that has been called crackling noise microscopy [73].

### Retrograde intrarenal surgery

With the increased use of RIRS as a technique for stone fragmentation, it is perhaps the fragmentation device most commonly used. Typical operating energies for these lasers far exceed the values of energies discussed in prior sections because higher energies will certainly cause fragmentation of the stone, however, they can also be associated with unwanted effects, such as an increased risk of damage to the mucosa or lining of the ureter and kidney, a risk of heating the irrigation fluid such that this might cause local damage to urothelial or surrounding tissues, and retropulsion of the stone. Parameters of the laser which are usually changed during fragmentation are laser energy, then frequency, and pulse width. There are also options dependent upon the laser type and manufacturer to alter the pulse shape, or to “modulate” the pulse. Alterations of any or all parameters may lead to greater or lesser efficiency of fragmentation. In weddellite stones, we expect the initiation of cracks by the laser will lead to propagating cracks without further intervention. The pulse frequency may play a role when pulse sequences are too rapid for the collapse mechanism to terminate after each pulse. The typical time scale for crack propagation to terminate is on the order of several microseconds (some MHz). Therefore, it would seem best to restrain the pulse frequency to lower frequencies. The high degree of tunability and popularity compared to other fragmentation techniques makes RIRS a suitable candidate for future AE experiments on kidney stone such as weddellite and uric acid which have been shown in this paper to fragment via avalanches. Targeting the low-energy failure regime shown in the prior section to instigate failure using RIRS would greatly impact the success rate and lower patient risk by limiting energy input of the laser.

### Percutaneous nephrolithotomy

With PCNL, stones are fragmented using various methods, including pneumatic lithotripsy, ultrasound or laser. Newer technologies have combined these methods, so that dual-energy probes which combine ultrasound and pneumatic lithotripsy are now available. Altering the frequency of the ultrasound or the frequency and displacement of the pneumatic device according to the stone type and avalanches seen may lead to more effective stone treatment.

### Shock wave generation lithotripsy

Shock wave generation SWL machines generate shock waves which propagate through a water medium to create a focused shock wave at the target site. The shock wave is characterised by its high amplitude, short duration and rapid rise time. When the shock wave encounters the stone, it creates a pressure differential across the stone’s surface. This pressure differential leads to the formation of stress waves and shear forces within the stone, causing microfractures and fragmentation. The created microfractures within the stone allow for the release of mechanical energy stored within the stone, leading to its disintegration into smaller fragments. There are several adjustable parameters which may be changed in order to maximise stone fragmentation efficiency in SWL. The energy level of the shockwave is one such crucial adjustable parameter. It can be modified by altering the electrical discharge intensity, duration, and focal length. Higher energy levels can effectively fragment larger stones but may increase the risk of tissue injury and discomfort. Conversely, lower energy levels may be used for smaller or softer stones and reduce the risk of complications. The focal size and depth can be adjusted to target the stone precisely. Smaller focal sizes improve targeting accuracy, while depth adjustments ensure that the shockwave focuses directly on the stone. Careful calibration of these parameters minimises collateral damage to surrounding tissues. Finally, the frequency of shock wave generation can affect the efficacy of stone fragmentation, with slower frequency leading to more efficient fragmentation.

### Extracorporeal shock wave lithotripsy

Again, knowledge of stone type prior to such treatment, and findings from further research investigating avalanches in different stone types, could help to optimise settings for ESWL. For example, we have shown that there is one dominant failure mechanism in the cystine stones which occurs on very short time scales. Therefore, it may be possible to generate shock waves using the lithotripter with higher frequencies and lower wavelengths to insight the failure mechanism present in all cystine stones. Additionally, weddellite stones which are calcium based can be brought back into the conversation when using ESWL. ESWL may have even greater success rate on the weddellite stones compared to the cystine stones because the weddellite stones have many failure mechanisms over a wide range of energies as shown in the prior section, making the avalanche regime an easier target.

### Future work

It is possible to think that further understanding of mechanisms of stone collapse might help us not only optimise the parameters of the lithotripsy device we use, but also determine which treatment or which device is likely to be most effective.

We have shown that the weddellite and uric acid stones contain some low-energy failure mechanisms and are power law distributed. As the collapse mechanisms are very different, the local heating will destroy the stones with different efficiencies which will need to be considered in experiments using various lithotripsy devices depending on which setting is being modified.

Several advances can now be contemplated. First, smaller detectors can be developed and used in in situ experiments. The most advanced technology is to use AFM needles as indenters to determine the avalanche spectrum [73].

These experiments need to be repeated across other stone types, such as whewellite, brushite and magnesium ammonium phosphate stones, with further analyses of samples of all stone types to improve accuracy. An understanding of the mechanical properties of mixed stone types should also be undertaken. Finally, ex vivo experiments using a laser and ultrasound or ballistic devices at different settings while monitoring for avalanches in various stones will assist us in determining the maximal efficiency settings for fragmentation.

Further research extended to all stone types, and measurement of stone responses to different lithotripsy strategies, will assist in optimisation of settings of the laser and other lithotripsy devices to treat stones.

## Conclusions

Under external load, kidney stones emit acoustic signals like crackling noise. The variability of the crackling noise is surprisingly great between different stone types, indicating different mechanisms of stone fracture. Two types of signals were found in all stones. At high energies of the emitted sound waves, we found “wild” avalanche behaviour, while all stones also show signals of “mild”, local, uncorrelated collapse. The key observation is that the crossover from mild to wild collapse events differs greatly between different stones. Weddellite shows brittle collapse where after some first impact the cracks propagate and the crossover is typically at energies below 5 aJ and stable over $\sim$6 orders of magnitude. In cystine and uric acid stones, the collapse is more complicated, with a dominance of local “mild” breakings. The cystine stones showed a narrow window of wild avalanche behaviour at high energies ($\sim$ 750 aJ), however, showed no signs of a lower energy wild avalanche regime that can be accurately described by a power law. The uric acid stones showed crossover points of $\sim$ 250 aJ and an avalanche regime which was stable over $\sim$ 3 orders of magnitude. Reproducing experiments using specific lithotripsy techniques on all stone types will provide the appropriate settings for energy to cause failure at significantly lower energies than is currently used in hospitals by targeting the wild avalanche regime reported in this paper, at energies greater than the crossover energy.

## Figures and Tables

**Table 3:**
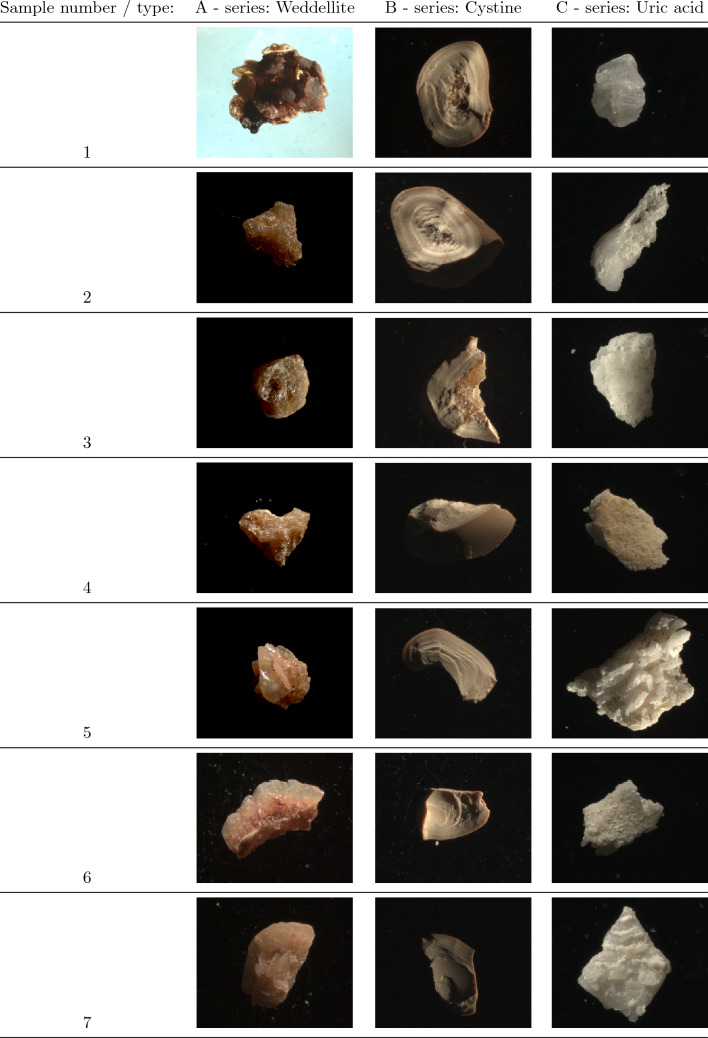
Kidney stone fragment sample images taken at 1.25$\times$ on an optical microscope

The second column shows A1–A7 weddellite class, the third column shows B1–B7 cystine class, and the fourth column shows C1–C7 uric acid

**Fig. 17:**
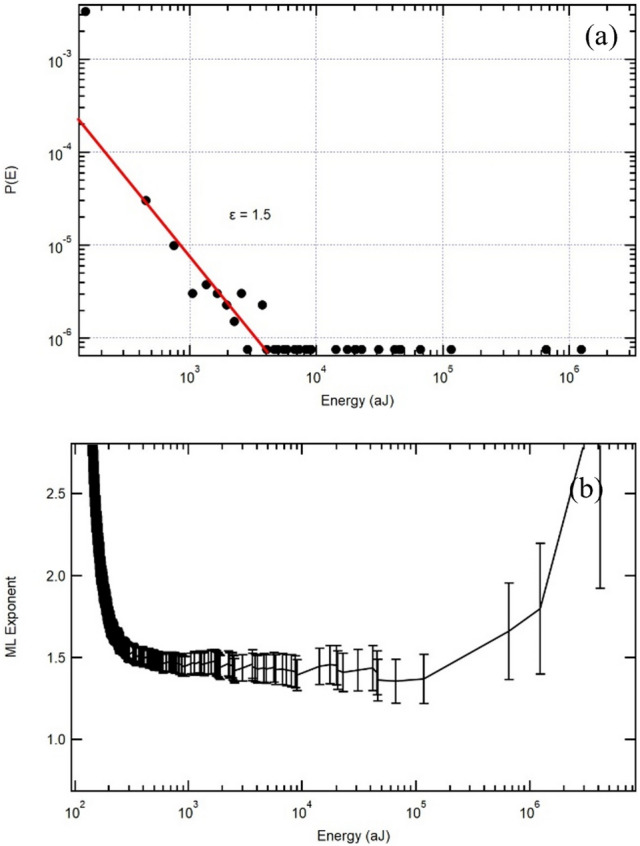
Energy PDF (**a**) and ML (**b**) for sample C2 revealing an energy exponent of $\epsilon =1.5$ stable over nearly the entire energy spectrum from 100 to 1,000,000 aJ

**Fig. 18:**
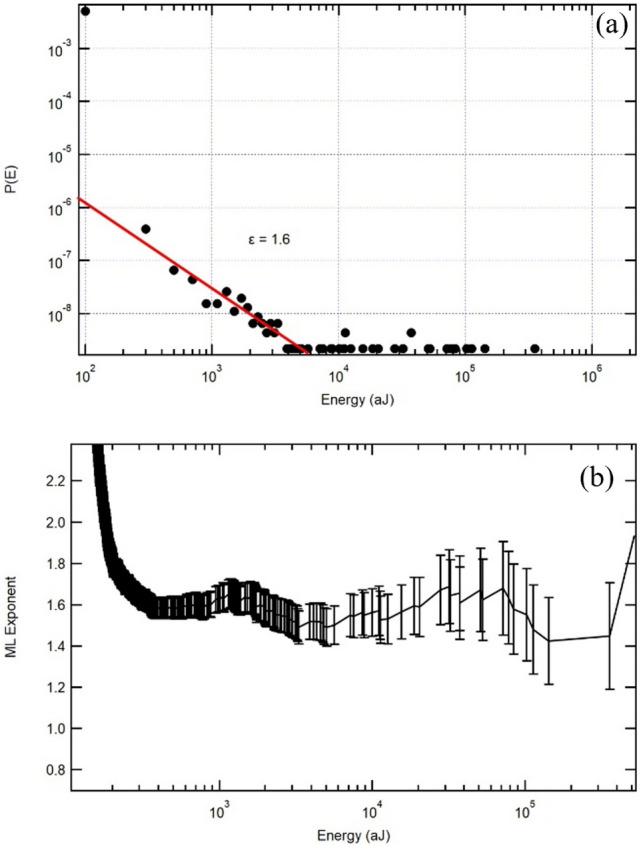
Energy PDF (**a**) and ML (**b**) for sample C3 revealing an energy exponent of $\epsilon =1.6$. There is a small hump in $\epsilon$ between 400 aJ and 1200 aJ and a decrease in $\epsilon$ from 1.6 to 1.4 at   $10^5$ aJ

**Fig. 20:**
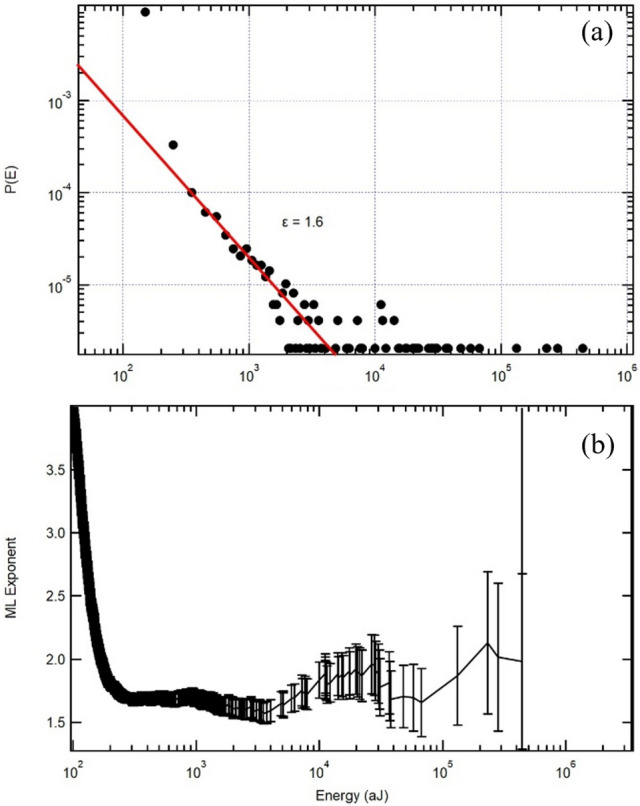
Energy PDF and ML for sample C5 revealing energy exponent of $\epsilon =1.6$

**Fig. 21:**
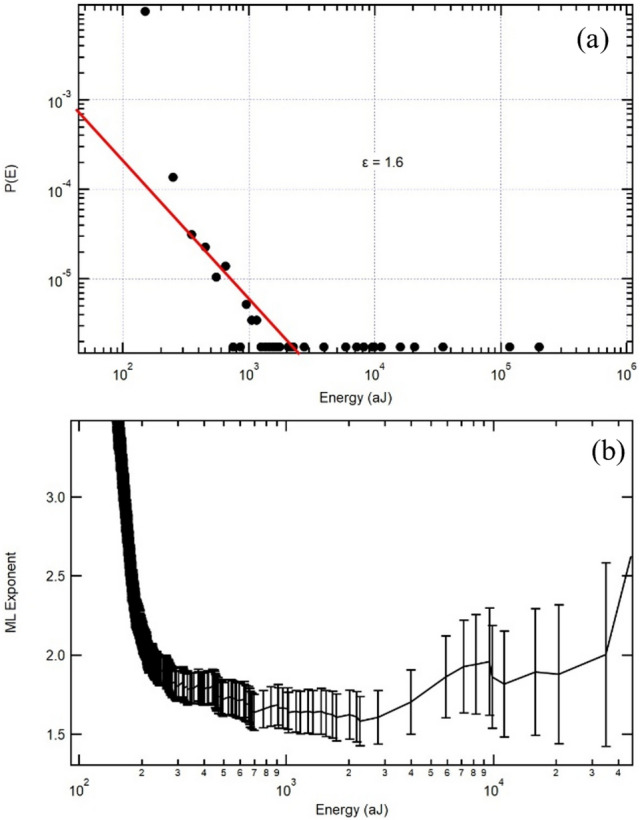
Energy PDF (**a**) and ML (**b**) for sample C6 revealing energy exponent of $\epsilon =1.6$

## Data Availability

The authors declare that the data supporting the findings of this study are available within the paper, its appendix, or its supplementary information files. The data that support the findings of this study are also available from the corresponding author upon reasonable request.

## Appendix A: Catalogue of kidney stone samples, energy PDF, and MLs

See Table 3 and Figs. 9, 10, 11, 12, 13, 14, 15, 16, 17, 18, 19, 20, 21 and 22 .
